# The Role of Immune Cells in the Pathogenesis of Idiopathic Inflammatory Myopathies

**DOI:** 10.14336/AD.2020.0410

**Published:** 2021-02-01

**Authors:** Lijuan Zhao, Qi Wang, Bin Zhou, Lihua Zhang, Honglin Zhu

**Affiliations:** ^1^Department of Rheumatology, Xiangya Hospital, Central South University, Changsha, Hunan, China.; ^2^Department of Radiology, Hunan Provincial People's Hospital and The First Affiliated Hospital of Hunan Normal University, Changsha, Hunan, China.; ^3^Department of Nephrology, The Affiliated Hospital of Qingdao University, Qingdao, China.; ^4^Department of Rheumatology, Hunan Provincial People's Hospital and The First Affiliated Hospital of Hunan Normal University, Changsha, Hunan, China.

**Keywords:** Idiopathic inflammatory myopathies, immune cells, pathogenesis

## Abstract

Idiopathic inflammatory myopathies (IIMs) are chronic autoimmune disorders involving multiple organs, such as the muscle, skin, lungs and joints. Although the detailed pathogenesis of IIMs remains unclear, immune mechanisms have long been recognised as of key importance. Immune cells contribute to many inflammatory processes via intercellular interactions and secretion of inflammatory factors, and many studies have demonstrated the participation of a variety of immune cells, such as T cells and B cells, in the development of IIMs. Here, we summarise the current knowledge regarding immune cells in IIM patients and discuss their potential roles in IIM pathogenesis.

## 1. Introduction

Idiopathic inflammatory myopathies (IIMs) comprise a group of heterogeneous rheumatic diseases predominantly characterised by progressive muscle weakness and reduced muscle endurance [1-4]. In addition to muscles, multiple other organs, such as the skin, lungs and joints, may be involved. The main subgroups of IIMs include dermatomyositis (DM), polymyositis (PM), inclusion body myositis (IBM) and immune-mediated necrotising myopathy (IMNM) [5, 6]. Treatment with high doses of glucocorticoids combined with immunosuppressive agents has largely improved the prognosis of IIMs, yet a considerable proportion of patients respond poorly and develop sustained muscle weakness [7, 8]. In general, a comprehensive understanding of IIM pathogenesis is needed for optimal therapeutic intervention.

Over the past decade, researchers have mainly focused on immune mechanisms in the development of IIMs due to the evidence regarding quantitative as well as qualitative abnormalities of immune cells in tissues and in the circulation for almost all types of IIMs [9, 10]. Lymphocytes are an important class of immune cells that are subdivided into T cells, B cells and natural killer (NK) cells. T cells and B cells are major components of adaptive immunity, recognising specific antigens and generating specific cell-mediated or antibody-mediated responses [11]. NK cells participate in innate immunity and function by releasing immunomodulatory cytokines or cytotoxic granules following their activation [12]. Other innate immune cells mainly include dendritic cells and macrophages, which initiate and support local immune responses by processing and presenting antigens [13]. In addition, mesenchymal stem cells and low-density granulocytes exhibit unique immunologic functions [14, 15]. Here, we discuss recent insight into the possible roles of these immune cells and highlight their intrinsic value as therapeutic targets in IIMs.

## 2. T cells in IIMs

T cells complete their primary development in the thymus and then enter the recirculation between the bloodstream and secondary lymphoid tissues [16]. Before they encounter specific antigens, mature recirculating T cells are known as naïve T cells. Once stimulated by antigen-presenting cells in a human leukocyte antigen (HLA)-dependent manner, T cells proliferate and differentiate into effector T cells or memory T cells [17]. There are several functional classes of effector T cells, which are specialised for different activities. CD8+ T cells recognise antigen peptides presented by HLA class I molecules, and their effector counterparts cytotoxic T cells (CTLs) have the ability to kill target cells [18]. After identifying the peptide:HLA complex on the surface of antigen-presenting cells, CD4+ T cells generate a flexible repertoire of effector subsets, including T helper (Th) cells, T follicular helper (Tfh) cells and regulatory T cells (Tregs); Th cells and Tfh cells activate target cells, whereas Tregs have a substantial immunosuppressive function [19].

T cells of diverse lineages have been implicated in the pathogenesis of IIMs [20]. CTLs produce toxic granules called perforins and granzymes, causing injury to myofibers that express morbidly increased amounts of HLA class I molecules [21, 22]. These CD8+ T cells often clonally expand in both diseased muscles and peripheral blood [23-26]. Additionally, Th cells orchestrate amplifying immune responses by secreting various cytokines [27, 28] and assist in antibody production by providing costimulatory signals [29]. Tregs inhibit the lytic activity of myo-reactive CD8+ cells and are proposed to play a role in counterbalancing muscle inflammation [30]. In a previous study, indirect proof of complete remission with renewal and diversification of the Treg compartment in two juvenile DM patients treated with autologous hematopoietic stem cell transplantation (HSCT) was obtained [31]. Furthermore, some special subtypes of T cells induce the breakdown of immune tolerance and further initiate the development of autoimmunity.

### 2.1 Muscle-infiltrating T cells

Because normal muscles rarely show immune cell infiltration, there have been efforts to elucidate how these cells migrate into muscles in IIMs. For example, chemokines produced by existing inflammatory cells or muscle fibres have been suggested to be involved in sustaining the trafficking of these cells [32]. Additionally, disrupted vascular permeability resulting from the deposition of complement membrane attack complexes [33], cleavage of endothelial VE-cadherin by up-regulated neutrophil serine proteinases [34, 35] and other factors can facilitate immune cell invasion. It is currently accepted that CD8+ T cells are more abundant near the endomysium in PM and IBM but that CD4+ T cells are more common in perivascular regions around the perimysium in DM [33].

#### CD28null T cells

Naïve T cells express CD28 as a costimulatory molecule for antigen-driven activation and proliferation [36, 37]. The frequency of CD28null T cells increases with the progressive differentiation of central memory T cells due to repeated antigen stimulation, although the mechanism has yet to be clarified [38, 39]. *In vitro* experiments indicate that TNF-α down-regulates T cell CD28 expression at the transcriptional level by inhibiting the activity of its minimal promoter [40]. A reduction in CD28 expression is thought to cause rapid immune responses independent of interactions with professional antigen-presenting cells when re-challenge with the same pathogen occurs [41]. In comparison with conventional CD28+ T cells, CD28null T cells are hypersensitive and can release large amounts of cytokines as well as cytolytic granules [42, 43]. Moreover, CD28null T cells are compromised with regard to antigen receptor diversity, antigen-induced proliferation and replicative lifespan [44]. It is reasonable that more CD28null T cells accumulate in elderly individuals because these individuals have experienced more antigenic stimulation [38]. In various chronic inflammatory processes, including viral infections and autoimmunity (such as Graves’ disease, ankylosing spondylitis and rheumatoid arthritis), CD28null T cells show expanded populations [45, 46] and confer cytotoxicity towards affected tissues [47].

CD28null T cells are increased in the muscle as well as the circulation of DM/PM patients, especially those seropositive for human cytomegalovirus. As the dominating T cell subsets of muscle infiltrates, the existence of CD28null T cells may be traced back to the time of diagnosis, and these cells persist during later disease stages [48]. High frequencies of CD28null T cells have also been found in muscle infiltrates and the circulation of IBM patients. Compared with peripheral blood, higher frequencies of CD28null T cells are present in inflamed muscle, indicating active recruitment, local proliferation or preferential retention of CD28null T cells in the tissue. Although these CD28null T cell populations are restricted in their T cell receptor (TCR) V_β_ usage, they are functionally non-senescent, with high interferon (IFN)-γ secretion and degranulation potential [49]. In PM, CD28null T cells, either CD4+ or CD8+, are capable of inducing a greater degree of muscle cell death than are their CD28+ counterparts[50]. To some extent, this may be attributed to polarised perforins because suppression of this process obviously reduces CD28null T cell cytotoxicity. In addition to perforins, the high level of IFN-γ secreted by CD28null T cells robustly up-regulates HLA (both class I and II) in muscles. Furthermore, interactions between TCR and HLA are required for the activation of CD28null T cells [42, 51], indicating the persistent destructiveness of CD28null T cells towards muscle fibres through a positive feedback loop. Interestingly, myotubes present greater sensitivity to CD28null T cell lethality than do myoblasts, possibly owing to muscle-specific antigens during differentiation [50]. Another characteristic of CD28null T cells is their anti-apoptotic property. Specifically, the proliferation and function of CD28null T cells are only partly suppressed by glucocorticoids and Tregs in DM/PM patients [52], which may be explained by the imbalance of anti-apoptotic proteins and pro-apoptotic molecules [53, 54]. For this reason, CD28null T cells persist in muscle tissue in many patients with DM/PM, who regain less than 75% of functional index even while responding well to conventional immunosuppressive treatment. In addition, a negative correlation between post-treatment CD28null T cells and comparatively poor outcomes has been validated [52].

#### Highly differentiated cytotoxic T cells

The refractoriness of some IIM patients towards corticosteroids and immunosuppressive agents hints at a specific nature of autoimmunity that conventional therapies cannot address [55]. In a genome-wide study of gene expression comparing muscle samples from IBM and other myopathies, a signature of highly differentiated cytotoxic CD8+ T cells (effector memory cells (TEMs) and terminally differentiated effector memory cells (TEMRAs)) was identified. Killer cell lectin-like receptor G1 (KLRG1), a marker of this population of cells, co-expresses with cytotoxic genes, and KLRG1+ cells were found to be abundant in IBM muscle, with a pattern of multifocal myofiber infiltration and invasion. Their circulating counterparts also account for a higher proportion of lymphocytes and CD8+ T cells in the peripheral blood of IBM patients [56]. These findings are in line with previous studies on CD28null T cells because T cells lacking CD28 are antigen experienced and highly differentiated [38].

#### T cells with special surface molecules

CD4+ and CD8+ T cells exhibit unique phenotypes depending on the expression of surface markers. CD45RO and CD45RA are two different isoforms of CD45. When expressed by T cells, CD45RO indicates antigen priming, whereas CD45RA represents an unprimed status [57]. CD45RO+ T cells, but not CD45RA+ T cells, predominate in DM/PM perivascular accumulation and in IBM/PM endomysial accumulation, and possible participation of these cells in muscle pathogenesis through an enhanced capacity of transendothelial migration or virgin-to-memory conversion occurring after diapedesis has been proposed [58]. NKG2D (natural-killer group 2, member D) is mainly expressed on the surface of NK cells. In a minority of cases, CD8+ T cells express NKG2D and gain the capacity to directly target cells independent of TCR. Chronic stimulation by IL-15 is a key mechanism in the acquisition of this capacity [59, 60]. In PM, muscle cells show predominantly higher expression of the NKG2D ligand MICA/B and membrane-related IL-15. *In vitro*, myoblast (treated with IFNγ and TNFα)-derived IL-15 is able to convert naïve CD8+ T cells into highly activated cytotoxic CD8+NKG2Dhigh T cells and maintain a pro-inflammatory environment [61]. Inducible co-stimulator-ligand (ICOS-L) belongs to the B7-family and is usually expressed on monocytes or dendritic cells. After interacting with its receptor ICOS on T cells, ICOS-L promotes Th1 and Th2 responses [62, 63]. ICOS-L was found to be abnormally expressed by IBM myofibers, and a class of autoinvasive CD8+ T cells expressing ICOS in inflamed muscles was detected. Moreover, ICOS·ICOS-L co-stimulatory signalling is associated with up-regulated effector perforin levels and may reflect the degree of endomysial inflammation [64]. CCR7 is the receptor for the chemokine CCL19, and both are very important in the homing and priming of T cells [65]. Without inflammation, the chemokine system is seldom expressed in muscles, but in PM and IBM, CCR7+ T cells preferentially surround and invade nonnecrotic muscle fibres positive for CCL19 [66, 67]. CXCL12 is another chemokine that acts as a ligand for CXCR4, and it has been suggested that their interaction helps recruit stem cells to tissues in need of repair [65]. A significantly high frequency of IL-4-producing CXCR4+ Th2 T cells has been documented in the muscles of DM/PM patients. Consistently, a high level of CXCL12 expression is observed in the vascular endothelial cells of muscle tissue. Furthermore, the number of CXCR4+ T cells correlates inversely with serum creatinine kinase (CK) and lactate dehydrogenase (LDH) levels [68]. These findings suggest possible roles for the above surface molecules during myositis progression, even though the mechanism and downstream events remain to be elucidated.

#### Biased cytokine expression reflecting T cell responses in muscles

Cytokines have important functions in inflammatory processes. Local release of T cell-derived cytokines can be regarded as a reflection of T cell responses. For instance, a higher count of IFN-γ-producing cells than IL-17-producing cells in the muscles of patients with DM/PM has been reported, and the ratio of IFN-γ/IL-17-producing cells is connected to the therapeutic results of high-dose immunoglobulin. Because IFN-γ and IL-17 are key cytokines for Th1 and Th17 cells, a reasonable inference about biased T cell subgroups was concluded [69].

### 2.2 Circulating T cells

It remains unclear whether muscle-infiltrating lymphocytes are activated *in situ* or in the circulation. On the one hand, studies have demonstrated the presence of extranodal lymphoid microstructures in muscle biopsy samples from patients with juvenile DM [70]; in this situation, lymphocytes are able to activate and differentiate in local lesions. On the other hand, circulating T-cell repertoires in IIMs are also dramatically perturbed, persistent in the long term and myocytotoxic [71, 72]. One typical example is the enrichment of highly pro-inflammatory and cytotoxic CD28null T cells found in the circulation of IIM patients [48, 49]. The similar nature of T cells in the two sites suggests their likely identical origin and underlines the particular clinical value of assessing these subsets in the peripheral blood.

#### Tregs

Although existence of Tregs in IIM muscle has been detected [30, 73], more attention has been paid to their circulating compartment, and a decreased percentage of Tregs in the peripheral blood of IIM patients has been reported. Among studies to date, the suppressor function of Tregs was normal or impaired [74-76]. The possible explanation for reduced Tregs levels is inappropriate apoptosis in these cells. Combined therapies using glucocorticoids plus antimalarial or other immunosuppressants might additionally diminish Treg proportions through inhibition of cytokines essential for cell homeostasis [74]. In juvenile DM, the normal percentages of peripheral Tregs are increased after high corticosteroid treatment. Nonetheless, Tregs separated from the blood of patients with active disease display a compromised suppressive function towards effector T cells when compared with patients in clinical remission. Effects of the pro-inflammatory environment on Treg function and the resistance of effector T cells to Treg-mediated suppression have been suggested as possible mechanisms [77].

#### T-bet (T-box expressed in T cells)^+^ cells

T-bet is one of two major T-box transcription factors essential for the differentiation of CTLs from naïve CD8+ T cells [78]. An increased frequency of CD8+ cells, including TEMs and TEMRAs, with high levels of T-bet expression in the peripheral blood of IBM patients has been observed. Although these CD8+T-bet+ cells overexpress the senescence marker CD57, a group of activated CD8+T-bet+ CD57null cells expressing lower levels of CD28, CD27 and CD127 as well as higher levels of CD38 and HLA-DR has been identified in IBM. In addition, CD8+T-bet+ cells are reported to be a favourable diagnostic biomarker. When the frequency of these cells is > 51.5%, the sensitivity and specificity for distinguishing IBM patients from other myositis patients can reach 94.4% and 88.5%, respectively [79].

#### Skewing of T cell subsets detected in the peripheral blood

Subpopulations of T cells are mutually restrained to maintain the balance of the immune response, and a shift in T cells can have stimulatory effects on IIM pathogenesis. For example, DM patients have a unique T cell signature with increased CD4+ T cell and Th17 cell frequencies in the peripheral blood. Both DM and PM patients exhibit decreased circulating CD8+ central memory T-cells [80]. A significantly lower frequency of IL-4+ Th1/IFN-γ+ Th2 cells in the peripheral blood of active DM patients than in healthy controls has been reported, supporting the deflection towards Th2-mediated inflammatory processes [81]. Additionally, levels of Tfh cells, which are specialised to support B cell maturation in germinal centres, were markedly increased in an IMNM patient with HMGCR autoantibody positivity and subsequently declined after immunosuppressive therapy, with an improved clinical outcome [82]. The circulating memory compartment of Tfh, which is defined as CXCR5+CD4+ T cells, is profoundly skewed towards Tfh2 and Tfh17, as opposed to Tfh1 cells, in juvenile DM patients, and such altered overall Tfh cell differentiation has been linked to disease activity and the frequency of blood plasmablasts [83]. The same discovery about Tfh cells was verified in DM/PM. In this study, elevated Th17 cells and decreased Tregs within the context of lymphopenia were also detected [84].

#### Transcriptomics and TCR-mediated signalling pathways in IIM T cells

Whole-transcriptome analysis of peripheral blood CD4+ and CD8+ T cells in DM/PM patients has revealed different profiles. Compared with DM, ANKRD55 and S100B are highly expressed in CD4+ T cells in PM. And a total of 176 genes were found to be differentially expressed (44 genes up-regulated and 132 genes down-regulated) in CD8+ T cells in PM. Functional analysis suggested that these altered genes are involved in lymphocyte migration and regulation of T-cell differentiation [85]. In studies of the TCR-mediated intracellular signalling pathway, expression of signal transducer and activator of transcription (STAT), forkhead box transcription factor (FoxP3) and TCR-induced phosphorylated zeta-chain-associated protein kinase 70 (pZAP70) in CD4+ T cells of DM patients was found to be suppressed. Furthermore, STAT and pZAP70 expression in CD8+ T cells is elevated in PM. Increases in suppressor of cytokine signalling-3 (SOCS3) and decreases in interleukin 6 signal transducer (IL6ST) in CD4+ T cells of both DM and PM patients have also been reported [86]. In summary, different gene expression and related pathways were found in T cells of DM and PM, suggesting different functions during DM/PM pathogenesis.

## 3. B cells in IIMs

The development of B cells begins in the bone marrow, where a diverse repertoire of B cell receptors (BCRs) can be generated through multiple rearrangements of immunoglobulin genes [87]. After eliminating autoreactivity, B cells gain tolerance to self-antigens and migrate to the periphery [88]. The final stages of B cell maturation occur in the follicles of the spleen and require the TNF family member B cell activating factor (BAFF) as well as B cell receptor signals [89]. Similarly, naïve B cells undergo no antigenic stimulation; they usually need to interact with Th cells for activation and ultimately differentiate into antibody-secreting plasma cells and memory B cells under antigen stimulation [90].

Activation of B cells in IIMs can be deduced from the frequent records of myositis-specific (MSAs) or myositis-associated (MAAs) autoantibodies, with apparent associations with clinical phenotypes and potential pathogenicity of self-antigens in target tissues [91-93]. BAFF is also believed to be crucial for the production of autoantibodies [89]. Increased levels of serum BAFF in subsets of IIMs have been reported, especially for patients positive for anti-Jo-1 antibodies. Moreover, BAFF levels correlate significantly with the titer of anti-Jo-1 antibodies and decline with the administration of glucocorticoids[94, 95]. Expression of BAFF is also markedly increased in muscle fibres in the perifascicular area of DM patients. Correspondingly, the receptor for BAFF is strongly expressed by infiltrated inflammatory cells [96, 97].

### 3.1 Muscle-infiltrating B cells

Despite not being as common as T cells, muscle-infiltrating B cells in muscle biopsy samples from patients with IIMs are clonally diversified and display deviant immunoglobulin V_H_ gene repertoires, indicating chronic local antigen-driven humoral responses [98, 99]. Interestingly, the quantity of B cells and the formation of ectopic follicle-like structures in the muscles of DM patients have been associated with the level of cytokines or chemokines involved in lymphoid neogenesis as well as type I interferon-related immunity [100]. However, concentrated collections of germinal-centre-like B cell follicles are absent in the muscles of patients with myositis. This finding argues against their necessity for antigen-stimulated clonal maturation of B cells [101]. Plasma cells are also components of inflammatory infiltrates in muscle biopsy specimens of IIM patients. In fact, the proportion of plasma cells (defined as CD138 positive) is even higher in IIM muscle than that of B cells [102].

### 3.2 Circulating B cells

Circulating B cells also undergo great changes in the disease process of IIMs. Naïve B cells and memory B cells are more and less abundant, respectively, in patients with DM/PM and anti-JO-1 antisynthetase syndrome (a special type of IIM characterised by antisynthetase autoantibodies and frequent interstitial lung disease) [80, 103, 104]. In pre-treatment juvenile DM, highly proliferative immature transitional B cells undergo significant expansion, and their frequency is strongly associated with the type 1 interferon signature. Given that the proportion or absolute number of immature transitional B cells correlates positively with disease activity, these cells are presumed to have a role in the development of juvenile DM [105]. Despite the fact that the percentage of transitional B cells in DM does not differ from that in healthy controls, levels of these cells are obviously decreased after treatment, which is accompanied by an increase in memory B cells [103]. In another study, a distinctly elevated proportion of RP105-negative B cells in DM compared with PM and normal controls was discovered. The loss of surface RP105 has previously been shown to act as a sign of B cell activation [106]. Similar to Tregs, regulatory B cells (Bregs) have potent immunosuppressive activity. Furthermore, Bregs are dramatically reduced in DM patients and may have a relationship with myositis-specific autoantibodies, pulmonary interstitial fibrosis and global disease scores [107].

## 4. Other immune cells in IIMs

### NK cells

Scant but sufficient evidence indicates altered quantities and functions of NK cells in patients with IIMs. In antisynthetase syndrome, NK cells exhibit an intense and diffuse distribution in fibrotic areas of the lungs, despite their rarity in muscle specimens. Although the quantity of NK cells in the circulation of patients with active disease is unaffected, these cells acquire an enhanced capacity to produce granzymes and IFN-γ [108, 109]. In juvenile DM, NK cells have been found to be enriched in the affected muscles of untreated patients, especially those with a short disease duration [110]. Conversely, the frequency of circulating NK cells is reduced and displays a trend towards normalisation after the cessation of active disease. These NK cells in patients with juvenile DM are more highly activated and proliferative than are the NK cells of healthy controls [111].

### Macrophages

Entry of macrophages into muscle tissue has been verified in all types of IIMs, including IMNM, with a main distribution around the endomysium and perimysium [91, 112-114]. Proteomic profiling of muscle biopsies derived from sIBM patients has also revealed the macrophage scavenger molecule CD163 as a highly and relevantly expressed protein, suggesting significant involvement of macrophages in muscle pathology [115]. Interestingly, a large proportion of diffuse endomysial macrophage infiltrates has more often been found in juvenile IIMs with MSAs towards nuclear matrix protein 2 (NXP2) and transcriptional intermediary factor 1γ (TIF1γ) [116], indicating a correlation of serological subtype with histological features. Upon activation, macrophages shed CD163 molecules from their membrane and release them into the peripheral blood [117], and higher levels of soluble CD163 in the circulation have been observed in patients with PM/DM complicated with interstitial lung disease (ILD) than in those without ILD and healthy volunteers [118]. That is, activation of macrophages may be important in PM/DM-related ILD.

### Dendritic cells

There are two main types of dendritic cells (DCs) mediating distinct biological processes. Myeloid DCs are potent antigen-presenting cells of adaptive immunity, and plasmacytoid DCs play a vital role in innate immunity, specifically during viral infection [32]. Although plasmacytoid DCs can also be present in the biopsies of normal muscle, their absolute count is very small. Both myeloid DCs and plasmacytoid DCs have been found in the muscle tissues of DM/PM/IBM patients, with a predominance of plasmacytoid DCs in DM and myeloid DCs in PM/IBM (plasmacytoid DCs were defined as BDCA-2+ or CD4+CD83+CD123+ and myeloid DCs as BDCA-1+ after excluding CD19+ B cells) [119-121]. Because plasmacytoid DCs are the major source of type I interferons, it makes sense why DM exhibits a remarkable type I interferon signature [122]. The distribution of DCs (immature DCs were defined as CD1a+ and mature DCs as CD83+DC-LAMP+) in inflamed muscles resembles a pattern of infiltrating T cells with a perivascular location in DM and an endomysial location in PM [27]. Their accumulation in IIM muscle tissues can contribute to an ongoing immune-mediated reaction and further muscle damage.

### Mesenchymal stem cells

Mesenchymal stem cells (MSCs) have gained much attention as a safe and beneficial therapy against a wide array of chronic inflammatory diseases [123, 124]. Transplantation of allogeneic MSCs showed efficacy in a small-scale pilot study including 10 patients with drug-resistant PM or DM. These patients exhibited decreased serum creatine kinase and improved muscle strength [125]. The underlying therapeutic mechanisms of MSCs in IIMs are unknown, and their regenerative and multipotential properties as well as potent immunosuppressive activities may be plausible; further exploration is needed [126, 127].

### Low-density granulocytes

Low-density granulocytes (LDGs) were first identified in systemic lupus erythematosus and later in many other autoimmune, cancer and infectious diseases [15]. These cells have either pro-inflammatory or suppressive properties, and the former is regarded as the main contributor to their pathogenicity via excessive secretion of IFN and TNF-α [128]. Besides, LDGs can efficiently form neutrophil extracellular traps (NETs) [129], which contain abundant endocellular materials and are able to activate the immune system as autoantigens under certain conditions [130]. The percentage of LDGs in the peripheral blood mononuclear cells of DM patients was abnormally increased. Moreover, patients with DM complicated by ILD have higher levels of LDGs than do those without ILD. LDGs may accelerate the onset of ILD in DM patients by abnormal NET regulation [131].

**Table 1:** Immune cells in IIMs.

Immune cells	Change	Characters	IIM subtype and classification criteria	Ref.
Muscle-infiltrating T cells				
CD28-T cells	increased	more cytotoxic, drug resistance	DM^1^, PM^1^, IBM^4^	[48-50, 52]
highly differentiated cytotoxic T cells	increased	co-express cytotoxic genes and KLRG1	IBM^5^	[56]
CD45RO+ T cells	increased	--	DM^1^, PM^1^, IBM^7^	[58]
CD8+NKG2D^high^ T cells	increased	target cells independent of TCR	PM^2^	[61]
CD8+ICOS+T cells	increased	enhanced perforin expression	IBM^3^	[64]
CCR7+ T cells	increased	--	PM^7^, IBM^7^	[66, 67]
IL-4-producing CXCR4+ Th2 T cells	increased	correlated with lower level of CK and LDH	DM^1^, PM^1^	[68]
IFNγ+Th1/IL17+Th17 ratio	increased	response poorly to high-dose immunoglobulins	DM^1^, PM^1^	[69]
Circulating T cells				
Tregs	decreased	normal function	DM^1,2^, PM^1^, IBM^4^	[74-76]
	normal	compromised suppressive function towards effector T cells	juvenile DM^1^	[77]
CD8+T-bet+ cells	increased	favourable value as diagnostic biomarker	IBM^5^	[79]
CD4+ T cells	increased or normal	different TCR-mediated characteristic signalling pathway	DM^1^, PM^1^, juvenile DM^1^	[80, 86]
CD8+ central memory T-cells	decreased	--	DM^1^, PM^1^, juvenile DM^1^	[80]
IL4+ Th1/IFNγ+ Th2 ratio	decreased	--	DM^1^	[81]
CXCR5+CD4+Tfh2&Tfh17/Tfh1 ratio	increased	linked with disease activity and frequency of blood plasmablasts	DM^1^, PM^1^, juvenile DM^1^	[83, 84]
Th17 cells	increased	--	DM^1^, PM^1^	[80, 84]
Muscle-infiltrating B cells	increased	clonally diversified and forming ectopic follicle-like structures	DM^2,7^, PM^2,7^, IBM^4^	[98-101]
CD138+ plasma cells	increased	--	PM^2^, IBM^4^	[102]
Circulating B cells				
naïve B cell/memory B cell ratio	increased	--	DM^1^, antisynthetase syndrome^7^	[103, 104]
transitional B cells	increased	associated with type 1 IFN signature	juvenile DM^1^	[105]
	normal but decreased after treatment	--	DM^1^	[103]
RP105 negative B cells	increased	sign of activation	DM^1^	[106]
Bregs	decreased	related to MSAs, ILD and global disease scores	DM^1^	[107]
Muscle-infiltrating NK cells	increased	--	juvenile DM^7^	[110]
Circulating NK cells	normal	enhanced capacity to produce granzymes and IFN-γ	antisynthetase syndrome^7^	[108, 109]
	decreased	normalize after cessation of active disease	juvenile DM^1^	[110]
Muscle-infiltrating macrophages	increased	correlated with MSAs	DM^3,7^, PM^3,7^, IBM^6^, IMNM^7^, juvenile DM^1^/PM^1^	[91, 112-114, 116]
Muscle-infiltrating DCs	increased	--	DM^1,2^/PM^1,2^/IBM^4^	[27, 119-121]
MSCs	--	show efficient therapeutic effects	drug-resistant DM^1^/PM^1^	[125]
LDGs in PBMCs	increased	associated with ILD	DM^1^	[131]

1, Bohan/Peter criteria; 2, criteria of the 119th ENMC international workshop; 3, Dalakas’ criteria; 4, Griggs’ criteria; 5, Lloyd’s criteria; 6, criteria of the 188th ENMC international workshop; 7, criteria not mentioned Th, T helper; IFN, Interferon; Tfh, T follicular helper; Tregs, regulatory T cells; T-bet, T-box expressed in T cells; Bregs, regulatory B cells; DCs, dendritic cells; MSCs, mesenchymal stem cells; LDGs, low-density granulocytes; PBMC, peripheral blood mononuclear cell; KLRG1, killer cell lectin-like receptor G1; TCR, T cell receptor; CK, creatinine kinase; LDH, lactate dehydrogenase; MSAs, myositis-specific antibodies; ILD, interstitial lung disease; DM, dermatomyositis; PM, polymyositis; IBM, inclusion body myositis; IMNM, immune-mediated necrotising myopathy

## 5. Therapies targeting immune cells or related pathways

Because immune cells are dominant components in the pathogenesis of IIMs, a number of pharmacological treatments targeting them, and related pathways have emerged. These therapies have provided IIM patients more choices and significant improvement in prognosis.

### Conventional immunosuppressants

Mycophenolate mofetil (MMF) and calcineurin inhibitors are two types of conventional immunosuppressants used in IIM patients. MMF can impair the proliferation of T cells and B cells. In an open study enrolling seven patients with either refractory DM or PM, MMF plus intravenous immunoglobulin (IVIG) led to complete remission in all cases [132]. The effectiveness of MMF alone or in combination has also been verified in several small case series [133-135]. Calcineurin inhibitors such as ciclosporin and tacrolimus can inhibit T cell activation and are commonly used in IIMs. Cyclosporine combined with glucocorticoids improved the findings of pulmonary function tests (PFTs) and chest high-resolution computed tomography (HRCTs) in 14 DM patients with acute/subacute ILD within 12 days from diagnosis [136]. Tacrolimus also showed favorable effects in treating 13 patients with antisynthetase syndrome (12 with anti-Jo-1 and 1 with anti-PL-12 autoantibodies), as indicated by better muscle strength, serum creatine CK levels, and PFT parameters [137]. In two more studies, one including 8 patients with myositis (6 with anti-Jo-1 and 2 with anti-SRP autoantibodies) and another including 31 patients with myositis (16 PM and 15 DM patients), tacrolimus improved both the muscle strength and serum CK levels [138, 139].

### Biologic and targeted synthetic disease-modifying anti-rheumatic drugs

Biological agents can selectively exhaust target immune cells or cytokines [140]. As a fusion protein composed of the Fc region of human IgG1 and the extracellular domain of cytotoxic T-lymphocyte-associated protein 4 (CTLA-4), abatacept inhibits the second signal for T cell activation by CD80 or CD86. In a randomised clinical trial, 42% of patients with refractory DM/PM achieved the preliminary definition of improvement coupled with increased Treg levels, as based on repeated muscle biopsies, after aggressive treatment of abatacept for 6 months [141]. Rituximab is able to rapidly deplete B cells by binding to the surface molecule CD20 and is promising for alleviating IIMs, even those with intractable phenotypes [142-146]. One review including 458 IIM patients who were nonresponsive to standard therapies found that 78.3% experienced improvement in at least one manifestation after treatment with rituximab. Among the cases, some were complicated with severe/refractory ILD [147]. Tocilizumab is an interleukin 6 (IL-6)-receptor antagonist with efficacy in treating IIMs as concluded in a few case reports [148, 149]. IL-6 can induce T cell differentiation and initially trigger BAFF [150]. However, more valid evidence from randomised, multicenter, double-blind trials is needed to confirm the curative effect of tocilizumab. Sifalimumab, an anti-IFNα monoclonal antibody, can interfere with the production of type I IFN-inducible proteins involved in the innate immune system. Treatment with sifalimumab in DM and PM patients resulted in suppression of the IFN signature in the blood and muscle tissue accompanied by clinical improvement [151]. The Janus kinase (JAK)- STAT signalling pathway is crucial in the IFN-mediated activation of cytokine receptors, and an inhibitor of JAK-1/3, tofacitinib, exhibited satisfactory effects on refractory DM as well as associated ILD [152-156].

## 6. Conclusion

Accumulating advances in pathogenesis elucidation have prompted a better understanding of IIMs. Immune cells in the peripheral blood and inflamed tissues of IIM patients show significant numerical or phenotypic changes leading to pathogenetic immune imbalance. The encouraging therapeutic effects of biologic agents targeting immune cells and related pathways also emphasise a key role for the immune response in the pathogenesis of IIMs. To our knowledge, this is the first review that summarises the participation of immune cells in IIMs and discusses potential targets for treatment. Because the specific approaches by which immune cells influence IIMs are still far from completely understood, elaborate mechanistic studies should be performed to facilitate more precise medical interventions.
